# Roles of the *MYB94/FUSED LEAVES1 (ZmFDL1)* and *GLOSSY2 (ZmGL2)* genes in cuticle biosynthesis and potential impacts on *Fusarium verticillioides* growth on maize silks

**DOI:** 10.3389/fpls.2023.1228394

**Published:** 2023-07-20

**Authors:** Giulia Castorina, Madison Bigelow, Travis Hattery, Massimo Zilio, Stefano Sangiorgio, Elisabetta Caporali, Giovanni Venturini, Marcello Iriti, Marna D. Yandeau-Nelson, Gabriella Consonni

**Affiliations:** ^1^ Dipartimento Di Scienze Agrarie e Ambientali (DiSAA), Università Degli Studi Di Milano, Milan, Italy; ^2^ Department of Genetics, Development, and Cell Biology, Iowa State University, Ames, IA, United States; ^3^ Dipartimento Di Bioscienze, Università Degli Studi Di Milano, Milan, Italy

**Keywords:** silk, maize, cuticle, cuticular wax, biotic stress, *Fusarium verticillioides*, *Fusarium* resistance, *Fusarium* ear rot

## Abstract

Maize silks, the stigmatic portions of the female flowers, have an important role in reproductive development. Silks also provide entry points for pathogens into host tissues since fungal hyphae move along the surface of the silks to reach the site of infection, i.e., the developing kernel. The outer extracellular surface of the silk is covered by a protective hydrophobic cuticle, comprised of a complex array of long-chain hydrocarbons and small amounts of very long chain fatty acids and fatty alcohols. This work illustrates that two previously characterized cuticle-related genes separately exert roles on maize silk cuticle deposition and function. *ZmMYB94*/*FUSED LEAVES 1* (*ZmFDL1*) MYB transcription factor is a key regulator of cuticle deposition in maize seedlings. The *ZmGLOSSY2* (*ZmGL2*) gene, a putative member of the BAHD superfamily of acyltransferases with close sequence similarity to the Arabidopsis *AtCER2* gene, is involved in the elongation of the fatty acid chains that serve as precursors of the waxes on young leaves. In silks, lack of *ZmFDL1* action generates a decrease in the accumulation of a wide number of compounds, including alkanes and alkenes of 20 carbons or greater and affects the expression of cuticle-related genes. These results suggest that *ZmFDL1* retains a regulatory role in silks, which might be exerted across the entire wax biosynthesis pathway. Separately, a comparison between *gl2-ref* and wild-type silks reveals differences in the abundance of specific cuticular wax constituents, particularly those of longer unsaturated carbon chain lengths. The inferred role of *ZmGL2* is to control the chain lengths of unsaturated hydrocarbons. The treatment of maize silks with *Fusarium verticillioides* conidia suspension results in altered transcript levels of *ZmFDL1* and *ZmGL2* genes. In addition, an increase in fungal growth was observed on *gl2-ref* mutant silks 72 hours after *Fusarium* infection. These findings suggest that the silk cuticle plays an active role in the response to *F. verticillioides* infection.

## 1 Introduction

The aerial surfaces of terrestrial plants are covered by the protective cuticle, a hydrophobic layer produced and secreted by the epidermis (Samuels et al., 2008), whose main function is to limit non-stomatal water loss and gas exchange with the external environment. The cuticle also mediates plant-environment interactions and protects against numerous abiotic and biotic stresses, including drought, ultraviolet radiation, and pest and pathogen invasion (Yeats and Rose, 2013; Ziv et al., 2018; Batsale et al., 2021).

The cuticle is comprised of the solvent-insoluble cutin polymer, which is a cross-esterified polymer of glycerol backbones and hydroxylated- and epoxy-long chain fatty acids of primarily 16 and 18 carbon atoms in length (Li-Beisson et al., 2013; Fich et al., 2016). This cutin matrix is embedded with intracuticular waxes and laid atop by epicuticular waxes, forming epicuticular films or wax crystalloids, depending on the epicuticular wax composition (Bernard and Joubès, 2013; Yeats and Rose, 2013). These cuticular waxes are a complex mixture of very-long-chain fatty acids (VLCFAs) of 20 or more carbon atoms in chain length, and their derivatives, which can include alcohols, aldehydes, alkanes, ketones and wax esters, as well as variable amounts of cyclic compounds, such as triterpenoids and phenylpropanoids (Bernard and Joubès, 2013; Lee and Suh, 2015a). This cuticle composition varies between plant species, among organs of a single plant, and across developmental stages. The cuticles of maize silks, which are the stigmatic portions of the maize female flowers, are particularly rich in simple long-chain hydrocarbons (e.g. n-alkanes, n-monoenes and n-dienes), and contain smaller amounts of long-chain fatty acids (LCFAs), aldehydes and alcohols (Yang et al., 1992; Perera et al., 2010; Loneman et al., 2017; Dennison et al., 2019). In contrast to the maize leaf cuticle, which does not accumulate alkenes, the silks contain a high proportion of alkenes (up to 55%) and low levels of alcohols (0-3%) compared to 15% of total cuticular waxes on leaves (Dennison et al., 2019). Moreover, the cuticular wax amount composition can vary along the silk length, particularly between the husk-encased portion of the silks and the portions that have emerged from husk encasement into the external environment. Specifically, wax concentrations accumulate up to five-fold higher on emerged as compared to husk-encased portions of silks (Loneman et al., 2017; Dennison et al., 2019). Furthermore, this difference is influenced by genotype, as previously shown across a panel of 32 inbred lines that showed an approximately 10-fold difference in cuticular wax accumulation on emerged silks. These differences in cuticular wax composition could be attributed to differences in environmental cues experienced by emerged and husk-encased silks as has been observed for other plant organs, including light, oxygen concentration and various biotic and abiotic stressors (Lewandowksa et al., 2020). A key regulator of cuticle deposition in seedlings is the *ZmMYB94*/*FUSED LEAVES1* (*ZmFDL1*) transcription factor (La Rocca et al., 2015; Castorina et al., 2020). Seedlings lacking ZmFDL1 show organ fusions, increased leaf permeability and altered distribution of epicuticular wax crystals (La Rocca et al., 2015; Castorina et al., 2020). Another gene shown to be important for cuticle composition and function is the *ZmGLOSSY2* (*ZmGL2*) gene, a member of the BAHD superfamily of acyltransferases (St-Pierre and De Luca, 2000; D’Auria, 2006). *ZmGl2* shares close sequence similarity to the Arabidopsis (*Arabidopsis thaliana*) *AtCER2* gene, and is involved in the elongation of the fatty acid chains (Tacke et al., 1995; Xia et al., 1996; Alexander et al., 2020) by a not yet defined mechanism. Lack of ZmGL2 in seedlings leaves confers the glossy phenotype, in contrast with the dull appearance of the wild-type leaves (Bianchi et al., 1975; Tacke et al., 1995), and results in altered distribution and morphology of epicuticular wax crystals (Beattie and Marcell, 2002). Both *ZmGL2* and *ZmFDL1*genes are highly expressed in silks (Sekhon et al., 2011; Sekhon et al., 2012; La Rocca et al., 2015), thus suggesting their involvement in controlling the deposition of silk cuticular lipids that are known to be present in large quantities on these tissues (Loneman et al., 2017).

The stigmatic silks of maize facilitate pollination by providing conduits for the elongating pollen tube, which transports the two sperm cells to the female gametes. However, during exposure to the external environment, silks also provide entry points for fungal pathogen infection of ovules and subsequently, developing kernels. Among these pathogens, *Fusarium verticillioides* is considered the main causative agent of Fusarium Ear Rot (FER) in maize (Thompson and Raizada, 2018). Although this fungus can grow inside the plant through several pathways, including passing from the roots to the stalk to the cob, or penetrating the ear through wounds produced by biotic or abiotic agents, the exposed portions of the silks were shown to be the most effective routes for *F. verticillioides* to naturally enter the ear (Koehler, 1942; Munkvold et al., 1997; Murillo-Williams and Munkvold, 2008). Indeed, *F. verticillioides* hyphae have been observed growing along the silk epidermis and invading the developing kernel at the bottom of the stylar canal (Duncan and Howard, 2010). Previous studies have demonstrated that *Fusarium* spore germination and growth in maize can be modulated by silk length and rates of senescence (Headrick et al., 1990; Miller et al., 2003). However, the genetic and molecular mechanisms underlying the silk-fungus interaction, and the role of the silk cuticle in mitigating fungal infection are not well investigated.

Control of fungal diseases with chemicals or agronomic approaches is often ineffective and increases production costs. For these reasons, genetic resistance to the fungal pathogen is the most durable and sustainable method to reduce losses (Lanubile et al., 2014). The identification of cuticle-mediated mechanisms involved in plant-fungus interactions would allow the selection of new genotypes that have characteristics of resistance to *Fusarium* spp. In this work, we have implemented our knowledge on the roles of *ZmFDL1* and *ZmGL2* in seedling cuticle deposition, by showing that these two genes are also involved in silk cuticle deposition. In addition, we have investigated *F. verticillioides* growth on silks of wild type and homozygous mutant plants.

To this aim, the cuticular wax composition and cuticle surface morphology were compared between silks from wild-type and *fdl1-1* and *gl2-ref* homozygous mutants. Furthermore, *F*. *verticillioides* development was compared for *fdl1-1* and *gl2-ref* mutant relative to wild-type silks following inoculation with a *F. verticillioides* conidia suspension to investigate cuticle-mediated responses to *F. verticillioides* development. This work demonstrates that *ZmFDL1* retains a regulatory role in silk cuticle deposition, while *ZmGL2* affects the abundance of only a few cuticular wax metabolites, particularly those of longer carbon chain lengths. The study of mutants in these genes provides evidence of the role exerted by silk cuticular waxes in mediating the response to *F. verticillioides* infection.

## 2 Materials and methods

### 2.1 Plant material and growth conditions

The previously described *fdl1-1* mutant (Castorina et al., 2020) used in this work was originally identified in the selfed progeny of a maize line crossed as female to an *En*/*Spm* line (La Rocca et al., 2015). The maize *gl2* mutant (*gl2-Salamini*, *gl2-ref*) seeds were obtained from the Maize Genetics COOP Stock Center (catalogue no. 208H; maizecoop.cropsci.uiuc.edu) (Portwood et al., 2019). The *gl2-ref* mutant allele was backcrossed one time into the H99 inbred line. The *fdl1-1* mutant allele was backcrossed three times into the B73 inbred line. In all the experiments performed, homozygous mutant and their wild-type control plants were from families segregating for *fdl1-1* or *gl2-ref*.

For *Fusarium* inoculation, wild-type and mutant seeds were germinated in a growth chamber under controlled conditions, 28/20°C temperature and 16h/8h day/night photoperiod, plants were transplanted and grown from mid-May until the end of July, up to the adult stage in an open field located in the Cascina Rosa Botanical Garden at the University of Milan, Italy. The plants were sown at a distance of 40 cm from each other in parallel rows spaced 70 cm apart. The experimental design was a randomized complete block arranged as a split plot with three replicates. The experiment was conducted for two consecutive years. For analyses of silk cuticle composition, segregating populations were grown following standard agronomic practice and without supplemental irrigation at the Iowa State University Ag Engineering and Agronomy Research Farm (Boone, IA). To prevent pollination, immature ear shoots were covered with Lawson #217 shoot bags.

### 2.2 Cuticular wax analysis

The cuticle composition of maize silks was analysed in populations segregating for *fdl1-1* and *gl2-ref*. Unpollinated ears were harvested three days after silk emergence from the encasing husk leaves, and were transported to the laboratory on ice. The husk-encased and emerged portions of the silks were collected and approximately 1 gram of fresh silk tissue per sample was extracted for 4 min in 9:1 hexane: diethyl ether, as previously described (Loneman et al., 2017). The extraction solvent was supplemented with 0.05 µg each of the internal standards C20 hydrocarbon, C17 alcohol, and C19 fatty acid per sample (Sigma-Aldrich, St. Louis, MO, USA). Extracts were dried under a stream of nitrogen gas and stored at -20°C. Extracts were then derivatized by silylation, dried under a stream of nitrogen gas, and resuspended in hexanes prior to gas chromatography (Loneman et al., 2017).

Gas chromatography was conducted using an Agilent Technologies (Santa Clara, CA, USA) model 6890A gas chromatograph (GC) coupled to either an Agilent 5975C triple-axis mass selective detector (MS) for the identification of metabolites or an Agilent model 6890 GC with Flame Ionization Detector (FID) for quantification following identification. The GC was equipped with a DB-1MS column (15 m x 0.25 mm x 0.25 µm). Samples were injected in splitless mode into an inlet maintained at 310°C, with a flow rate of 1.2 mL/min of helium carrier gas. In the case of GC-MS, the MS transfer line temperature was set to a constant 280°C with an ion source temperature of 340°C. For GC-FID, the ion source temperature was set to 250°C. For both instruments, injection of 1 µL of extract occurred an initial temperature of 80°C (solvent delay of 3 minutes), ramped to 220°C at 15°C/min, ramped to 308°C at 7.5°C/min, ramped to 340°C at 20°C/min, and held for 2.3 minutes.

Separated metabolites were identified by MSD ChemStation software (Agilent Technologies) and AMDIS software (Stein, 1999) using their retention index (RI), mass (m/z), and by comparison to the National Institute of Standards and Technology Mass Spectral Library (http://webbook.nist.gov/chemistry/). Metabolite abundances were calculated by relative quantification to the internal standard C20 hydrocarbon relative to the dry weight of the sample (µmol/g dry weight), as previously described (Loneman et al., 2017). The Limit of Quantification (LOQ) and Limit of Detection (LOD) were determined by injection of a serial dilution series of the external standard, nonadecane in triplicate. The LOQ and LOD for this study were 9.3E-9 and 9.3E-11 µmol, respectively.

### 2.3 Microscopy analyses

To obtain images of the epicuticular layer, emerged portions of the silks of wild-type and mutant plants were harvested four days post-emergence, dried and processed according to Castorina et al. (2018). Micrographs of silks were taken with a scanning electron microscope (ZEISS Sigma FE-SEM) at different magnifications to observe morphological differences between the analyzed genotypes.

### 2.4 Experimental inoculations of *Fusarium verticillioides*

The conidia suspension was prepared from *F. verticillioides* strains belonging to the fungi collection of the Department of Agricultural and Environmental Sciences of the State University of Milan. For the experimental inoculations, eight *F. verticillioides* strains (Fv2003, Fv2010, Fv2170, Fv2198, Fv2221, Fv2232, Fv2233, Fv2306) isolated from naturally infected maize kernels and able to produce fumonisins *in vitro*, belonging to the collection of the Mycology Laboratory at DiSAA, University of Milan were used. Strains were grown on plates with potato dextrose agar (PDA) and incubated in dark conditions for 7 days at 25°C. A conidia suspension for each strain was prepared in sterile water and 0.01% Tween20^®^ and were mixed in the same quantities to create a single suspension with a concentration of 1 x 10^6^ conidia/mL.

Maize ears were self-pollinated and after 7 days, at the beginning of silk browning (Schaafsma et al., 1997), they were inoculated with *F. verticillioides* by spraying the conidia suspension directly on the silks (**Supplementary Figure 1A**). An equal number of ears were inoculated with a mock solution and used as control. After inoculation, the ears were covered with transparent plastic bags (**Supplementary Figure 1B**) to avoid environmental *F. verticillioides* contamination and to promote the formation of high humidity conditions. For subsequent analyses, the whole silks were collected at 0 and 72 hours after the inoculation (**Supplementary Figure 1C**). At least three independent biological replicates were taken for each treatment. The samples were prepared by grinding the silks in liquid nitrogen with a mortar and pestle. The powdered material was stored at -80°C. For Fusarium Ear Rot symptom evaluation, ears were covered with a fine-mesh net bag to prevent insect damage and were grown to maturity (**Supplementary Figure 1D**).

### 2.5 Nucleic acid extraction, cDNA preparation and quantitative gene analysis

Genomic DNA extractions were performed as previously described (Gutiérrez-Marcos et al., 2007). Total RNA was extracted with the Rapid CTAB Protocol (Gambino et al., 2008). Nucleic acid purity and quantity were measured with the NanoDrop^®^ ND-1000 spectrophotometer (Thermo Scientific). Total RNAs were treated with RQ1 RNase-Free DNase (Promega, Switzerland) and first-strand cDNA was synthesized using the High-Capacity cDNA Reverse Transcription Kit (Thermo Fisher Scientific) according to the manufacturer’s recommendations. Genomic DNA and cDNA were subjected to amplification with GoTaq^®^ Flexi DNA Polymerase (Promega). Quantitative Real-Time PCR (RT-qPCR) was performed on cDNA or DNA with the 7300 Real-Time PCR System (Applied Biosystems), using GoTaq qPCR Master Mix (Promega), in a final volume of 10 μL. The relative transcript level of the maize genes was calculated by the 2^-ΔCt^ method (Livak and Schmittgen, 2001) using the expression of the *ZmEF1α* gene as a reference. Primers used are reported in **Supplementary Table 1**.

### 2.6 Quantification of fungal DNA

To quantitatively determine *F. verticillioides* growth, the fungal DNA was quantified amplifying the *polyketide synthase* gene (*FvFUM1*). For each sample, 20 ng of total DNA were analyzed by RT-qPCR as follows: 2 min at 95°C, followed by 45 cycles of 15 s at 95°C, 20 s at 65°C and 20 s at 72°C. Serial dilutions ranging from 20 to 2x10^-4^ ng/uL of *F. verticillioides* DNA extracted from lyophilized pure cultures were used to generate a standard curve resulting in an R^2^ of 0.99 (**Supplementary Figure 2**). The curve was used for DNA quantification. Final quantification results were expressed as $\frac{\text{fungal DNA}\left(\text{ng}\right)\;}{\text{total DNA}\left(\text{ng}\right)}$ ratio.

### 2.7 Assessment of Fusarium ear rot

Twelve mature ears inoculated with fungal suspension and twelve inoculated with the mock solution for each genotype were manually harvested. Harvested ears were de-husked by hand and the severity of Fusarium ear rot (FER) symptoms was evaluated using rating scales based on the percentage of kernels with visible symptoms of infection, such as rot and mycelium growth.

FER incidence was calculated as the percentage of ears with visible symptoms of the disease. The FER severity was measured on a visual rating scale of seven classes (0-6) based on the percentage of visibly infected kernels (0 = 0% infected kernels detected; 1 = 1–3%; 1 = 4–10%; 3 = 11–25%; 4 = 26–50%, 5 = 51–75%; 6 = 76–100%) (Reid et al., 1996) and then expressed as an index of infection using the modified Townsend–Heuberger formula (Townsend and Heuberger, 1943): FER= ∑ [(n x c)/6 N]x100, where n is the number of ears per class, c is the numerical value of each class, and N is the total number of ears examined per replicate.

### 2.8 Statistical analysis

All data were statistically analyzed using the Student’s t test (ns=not significant, *P< 0.05, **P< 0.01, ***P< 0.001 and ***P< 0.0001) or two-way analysis of variance (ANOVA) (*P*< 0.05) using the statistical packages Prism GraphPad 6 or JMP Pro statistical software version 16 (SAS Institute Inc., Cary, NC, 1989–2021).

## 3 Results

### 3.1 *ZmFDL1* and *ZmGL2* impact cuticle composition on maize silks

**Figure 1:**
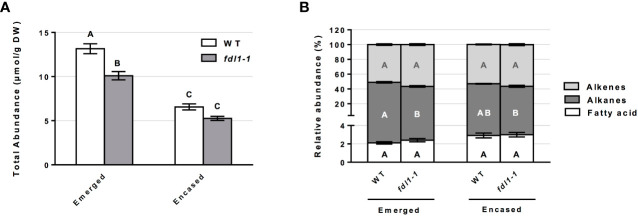
Cuticular wax abundance on silks of wild-type (WT) and *fdl1-1* mutant plants. **(A)** Total abundance of cuticular waxes estimated for the emerged and husk-encased portions of the silks. **(B)** Percentage of hydrocarbon (alkanes and alkenes) and long chain and very long chain fatty acid classes relative to total cuticular wax accumulation on emerged and husk-encased portions of silks. Values represent the mean of independent biological replicates for wild-type (emerged N=38; encased N=23) and fdl1-1 (emerged N=25; encased N=16) genotypes. Error bars are ± SE. Different letters above or within data-bars denote statistically significant differences between genotypes assessed by two-way ANOVA (*P*< 0.05). In panel **(B)** black, white and grey letters represent comparisons within a specific class of cuticular waxes.

To examine the impacts of the ZmFDL1 transcription factor on the silk cuticle, cuticular wax composition was separately profiled for the emerged and husk-encased portions of silks three days after the silks first emerged from the encasing husk leaves from wild-type and *fdl1-1* mutant plants. Total cuticular wax abundance was reduced by 23% on the emerged portions of silks and reduced by 20% on encased portions of silks from *fdl1-1* homozygotes as compared to wild-type (**Figure 1A**).

The accumulation of these cuticular wax metabolite classes can also be considered relative to total cuticular waxes. The examination of the relative abundances for the single class of cuticular hydrocarbons, in both husk-encased and emerged silks (**Figure 1B**), reveals that no statistical differences were detected for husk-encased silks from wild-type versus *fdl1-1* mutant plants. In contrast, a 6% statistically significant reduction in the relative abundance of alkanes was observed on emerged portions of silks from the *fdl1-1* mutant with concomitant, but non-significant increases in the relative abundances of fatty acids and alkenes.

**Figure 2:**
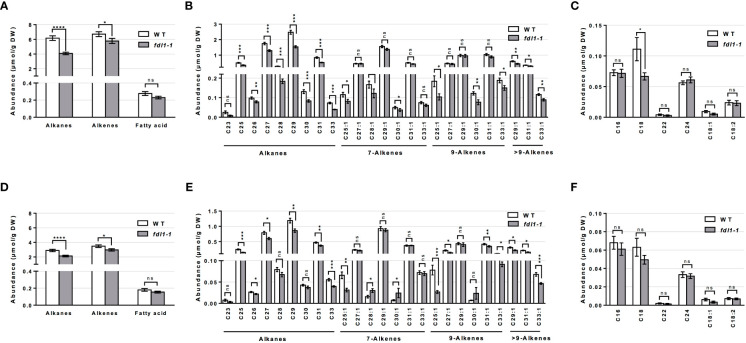
Cuticular wax composition on wild-type (WT) and *fdl1-1* mutant silks. **(A, D)** Abundance of cuticular waxes separated by class of metabolites, **(B, E)** abundance of individual cuticular hydrocarbon constituents and **(C, F)** abundances of individual cuticular wax long chain and very long chain fatty acid constituents on **(A-C)** emerged and **(D-F)** husk-encased portions of silks. Values represent the mean of independent biological replicates for wild-type (emerged N=38; encased N=23) and fdl1-1 (emerged N=25; encased N=16) genotypes. Error bars are ± SE. Asterisks denote significant differences between WT and *fdl1-1*, as assessed by Tukey’s HSD (* *P* ≤ 0.05; ** *P* ≤ 0.01; *** *P* ≤ 0.001; **** *P* ≤ 0.0001; ns, not significant).

Analyzing the total abundance of the cuticular hydrocarbons classes, we observed that in the *fdl1-1* mutant the alkanes were reduced by 34% and 26% on emerged and husk-encased silks, respectively (**Figures 2A, D**). The alkenes (i.e. unsaturated hydrocarbons) were reduced by 14% and 15% on emerged and husk-encased silks, respectively. Although not statistically significant, long chain and very long chain FAs (i.e. fatty acids ranging from 16 to 24 carbon atoms in chain length) were reduced by 17% and 14% on emerged and husk-encased silks, respectively (**Figure 2A, D**).

The abundances of the individual cuticular wax metabolites reveals that, in both husk-encased and emerged silks, a majority of individual saturated (alkane) and unsaturated (alkene) hydrocarbon metabolites accumulated to lower levels in the *fdl1-1* mutant as compared to wild-type (**Figures 2B, E**). The individual long chain and very long chain fatty acid constituents (**Figures 2C, F**) did not show any differences between genotypes with the exception of C18 FA, which accumulated to a statistically lower level on the emerged portions of the *fdl1-1* silks (**Figure 2C**). These results suggest that ZmFDL1 impacts the accumulation of cuticular hydrocarbons regardless of chain length or level of unsaturation.

**Figure 3:**
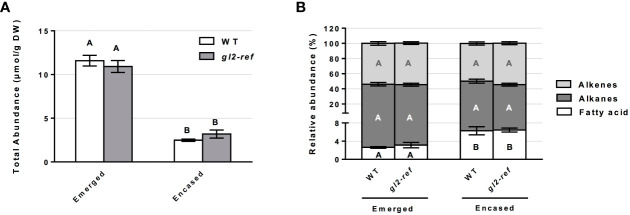
Cuticular wax abundance on silks of wild-type (WT) and *gl2-ref* mutant plants. **(A)** Total abundance of cuticular waxes estimated for the emerged and husk-encased portions of silks. **(B)** Percentage of hydrocarbon (alkanes and alkenes) and long chain and very long chain fatty acid classes relative to total cuticular wax accumulation of emerged and husk-encased portions of silks. Values represent the mean of independent biological replicates for wild-type (emerged N=8; encased N=7) and *gl2-ref* (emerged N=8; encased N=9) genotypes. Error bars are ± SE. Different letters above or within data-bars denote statistically significant differences between genotypes assessed by two-way ANOVA (*P*< 0.05). In panel **(B)** black, white and grey letters represent comparisons within a specific class of cuticular waxes.

**Figure 4:**
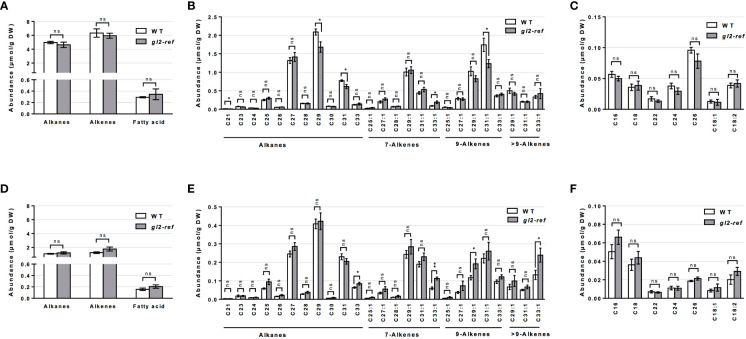
Cuticular wax composition in wild-type (WT) and *gl2-ref* mutant silks. **(A, D)** Abundance of cuticular waxes separated by class of metabolites, **(B, E)** abundance of individual cuticular hydrocarbon constituents and **(C, F)** abundances of individual cuticular wax long chain and very long chain fatty acid constituents on **(A-C)** emerged and **(D-F)** husk-encased portions of silks. Values represent the mean of independent biological replicates for wild-type (emerged N=8; encased N=7) and *gl2-ref* (emerged N=8; encased N=9) genotypes. Error bars are ± SE. Asterisks denote significant differences between WT and *gl2-ref*, as assessed by Tukey’s HSD (* *P* ≤ 0.05; ** *P* ≤ 0.01; ns, not significative).

Similar to the cuticular wax profiling of the *fdl1-1* mutant, cuticular wax composition was profiled on the emerged and husked-encased portions of silks from wild-type and *gl2-ref* mutant plants. Total cuticular wax accumulation did not statistically differ between *gl2-ref* and wild-type silks in both emerged and husk-encased portions of silks (**Figure 3A**). Similarly, there were no statistical differences in relative abundances of the silk cuticular wax classes out of total waxes between wild-type and mutant plants (**Figure 3B**). Differences in abundance were not observed for the metabolite classes of cuticular waxes between *gl2-ref* and wild-type silks (**Figures 4A, D**) but only for specific cuticular wax constituents, particularly those of longer carbon chain lengths (**Figure 4**). For emerged portions of silks, accumulation of the C21, C29 and C31 alkane and C31:1 (9) alkene were 28%, 15%, 20% and 29% higher, respectively in wild-type as compared to *gl2-ref*. In contrast, the C33:1 (7) alkene was 46% more abundant in the *gl2-ref* mutant as compared to the wild-type (**Figure 4B**). Similarly, for encased portions of silks from the *gl2-ref* mutant, the C33 alkane, the C33:1 (7), C29:1 (9) and C33:1 (>9) alkenes accumulated 33% 88%, 48% and 81% more highly, respectively (**Figure 4E**) as compared to wild-type. VLCFA metabolite abundances did not statistically differ between wildtype and gl2-ref plants for either emerged or encased portions of silks (**Figures 4C, F**). These data demonstrate that specific very-long-chain hydrocarbons accumulate to higher levels in the *gl2-ref* mutant and therefore that ZmGL2 contributes modestly to cuticular wax accumulation and composition. When ZmGL2 is not present the silks contain more C33:1 metabolites, suggesting that ZmGL2 may function to limit the chain lengths of unsaturated hydrocarbons.

The *ZmFDL1* product is a key regulator of cuticle deposition in juvenile vegetative tissues and the lack of its activity affects the expression of several cuticular wax genes involved in different modules of the biosynthetic network (Castorina et al., 2020). We previously reported that *ZmCER1*, *ZmCER4* and *ZmWSD11* cuticle-related genes were differentially expressed in *fdl1-1* young seedlings. The expression of these genes were next analyzed in silks (**Supplementary Figure 3**). Consistent with previous observations based on RNA-Seq data and quantitative expression analysis (Castorina et al., 2020), the *ZmCER1* gene was upregulated in silks of *fdl1-*1 plants as compared to control wild-type plants, while *ZmCER4* was downregulated. In contrast, the *ZmWSD11* gene, which was up-regulated in *fdl1-1* seedlings (Castorina et al., 2020), is not differentially expressed in *fdl1-1* silks as compared to wild-type.

### 3.2 Analysis of silk surface on wild-type and mutant silks

**Figure 5:**
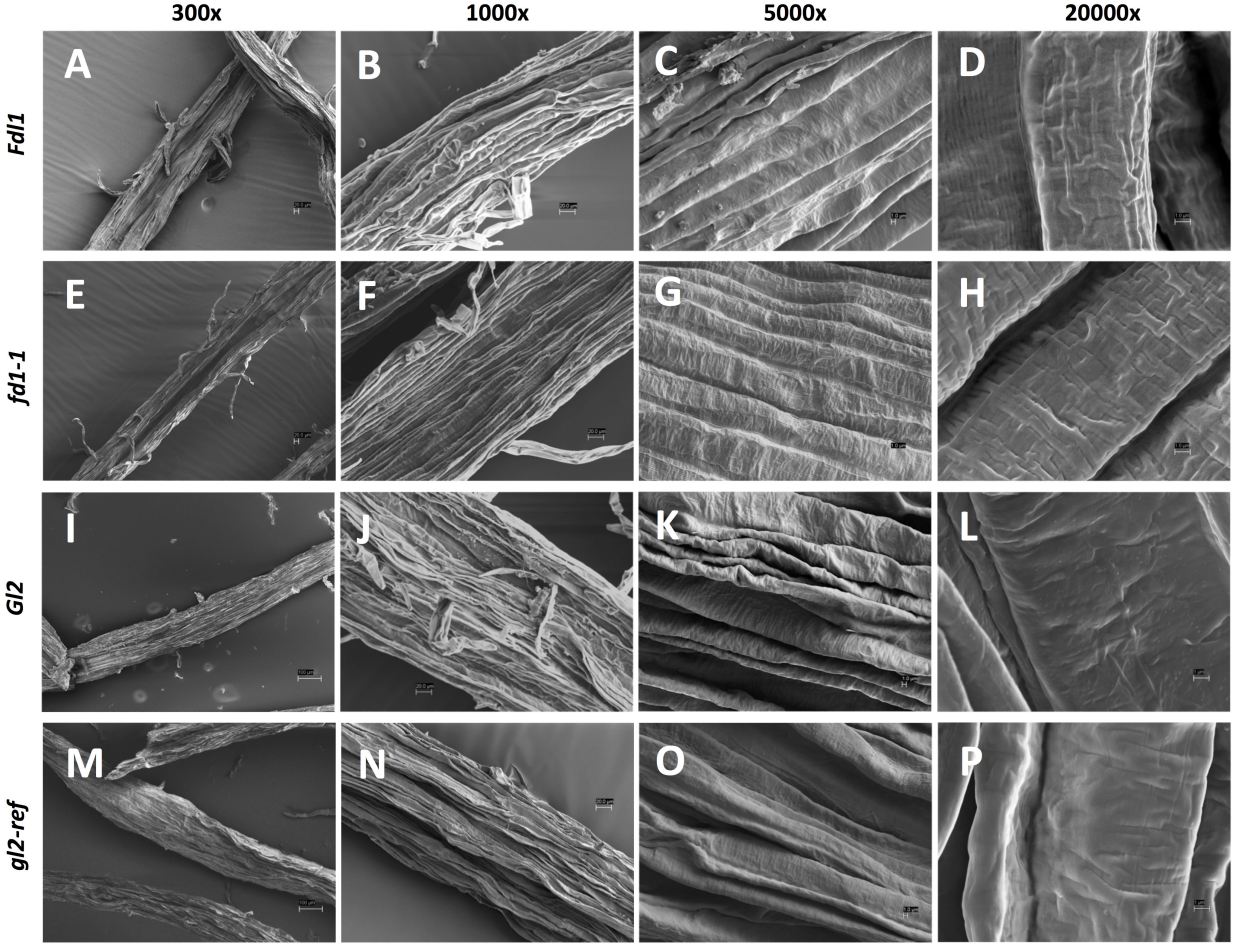
Scanning electron microscopy (SEM) micrographs of silk tissues. Images of the husk-emerged silk epicuticular layer of **(A-D)** *Fdl1* wild-type and **(E-H)** *fdl1-1* homozygous mutant plants, **(I-L)** *Gl2* wild-type and **(M-P)** *gl2-ref* homozygous mutant plants have been acquired at 300x, 1000x, 5000x and 20000x magnification. Scale bars of 100 µm (I,M), 20 µm **(A, B, E, F, J, N)** and 1 µm **(C, D, G, H, K, L, O, P)** are also reported.

Because variations in silk cuticular wax composition were detected at the biochemical level in each mutant, we examined whether epicuticular ultrastructure was impacted. Detailed analyses were conducted by scanning electron microscopy (SEM) on silks of wild-type, homozygous *fdl1-1* and *gl2-ref* mutant plants. Representative images of each genotype taken at different magnifications are shown in **Figure 5**; they provide an in-depth characterization of maize silk surface morphology and epicuticular wax distribution. At lower magnification (300x), the silk surface appears crenulated and trichomes are visible (**Figures 5A, E, I, M**). No differences were detected among wild-type and mutant genotypes. At higher magnification (5000x and 20000x), it was possible to analyze the patterns of epicuticular waxes distribution. We demonstrate that, differently from many other plant tissues on whose surface epicuticular waxes are deposited as crystalloids of different morphologies (Beattie and Marcell, 2002), on maize silks, epicuticular waxes are distributed as an amorphous film (**Figures 5D, H, L, P**). No evident morphological differences between wild-type and mutant plants were detected (**Figure 5**).

### 3.3 Cuticle-mediated response to *F. verticillioides* infection

Silks are the preferential routes of entry into the developing grains for mycotoxigenic fungi. *F. verticillioides* hyphae grow along the epidermis of silks, until they reach the developing kernels (Bacon et al., 2008; Duncan and Howard, 2010; Thompson and Raizada, 2018). To evaluate whether silk cuticle composition affects plant-pathogen interaction, we conducted experimental inoculation of *F. verticillioides* on the silks of WT, *fdl1-1* and *gl2-ref* plants with a control solution or conidial suspension and measured the fungal growth on silks at 0 and 72 hours after the inoculation. Silks were collected in their entirety (i.e. both emerged and husk-encased portions) at either time point and the growth of the fungus was evaluated through molecular quantification of its DNA. Fungus growth was assessed in silks collected for two consecutive years and results were consistent.

**Figure 6:**
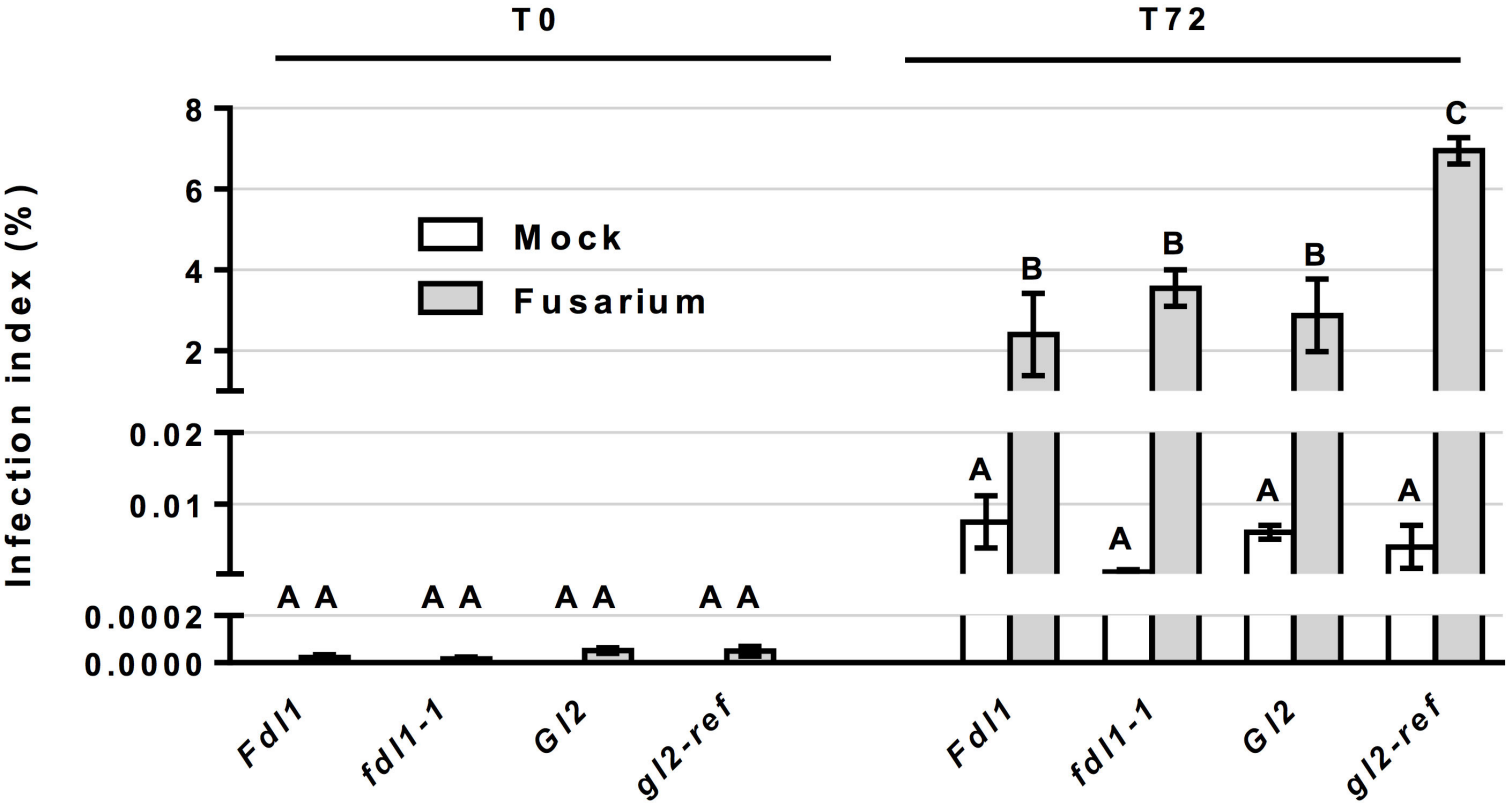
Molecular quantification of *Fusarium verticillioides* on silks. Quantification of fungus growth in control (Mock) and treated (Fusarium) silks of wild-type siblings (*Fdl1* and *Gl2*), *fdl1-1* and *gl2-ref* homozygous mutant plants, has been evaluated through the molecular quantification of *Fusarium verticillioides* DNA, by Real-Time PCR, at 0 and 72 hours after treatment. Values represent the average for a minimum of three independent biological replicates. Error bar ± SE. At T0, no discernable infection was detected in the Mock treatment and no statistical differences were detected between Mock and *Fusarium* inoculated silks. Different letters denote statistical significance assessed by two-way ANOVA (*P*< 0.05).

The molecular quantification of fungus growth performed in *fdl1-1, gl2-ref* mutant and wild-type silks indicated an extremely low level of fungus contamination in all mock-treated samples and did not show any differences among genotypes as compared between 0 and 72 hours after mock treatment (**Figure 6**). The growth capacity of the fungus was not impacted by the decreased cuticular wax abundance in the *fdl1-1* mutant, as evidenced by similar fungal growth patterns on *F. verticillioides* treated *fdl1-1* silks as compared to wild-type plants at 72 hours after inoculation. In contrast, in the *gl2-ref* mutant, although the total wax load was not affected, an increase in *F. verticillioides* growth at 72 hours after inoculation was observed (**Figure 6**). These results suggest that the changes observed for only a few specific cuticular wax metabolites (**Figure 4**) affect fungus growth on *gl2-ref* mutant silks.

**Figure 7:**
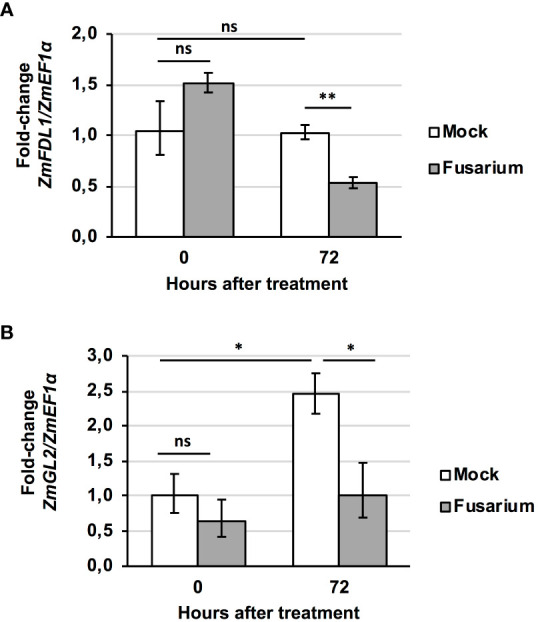
*Fusarium*-induced changes in gene transcript level. Expression profile of **(A)** *ZmFDL1* and **(B)** *ZmGL2* gene in silks of wild-type plants at 0 or 72 hours after treatment with control (Mock) or *Fusarium verticillioides* conidia suspension (Fusarium). Values represent the mean fold change of three independent biological replicates. Error bar ± SD. Significant differences were assessed by Student’s t-test (* *P* ≤ 0.05; ** *P* ≤ 0.01; ns, not significant).

We also evaluated variations in the transcript levels of *ZmFDL1* and *ZmGL2* genes in wild-type silks in the presence or in absence of the fungus at 0 and 72 hours (**Figure 7**). The *ZmFDL1* gene did not show changes in the transcript level in untreated plants but was down-regulated, compared to the control, at 72 hours after treatment with the conidial suspension (**Figure 7A**). The transcript of the *ZmGL2* gene appeared to be more abundant in control plants at 72 hours compared to time zero. Similarly, *ZmGL2* appeared to be down-regulated in treated plants, compared to untreated control plants, 72 hours after spraying the conidial suspension of *Fusarium* (**Figure 7B**).

Kernel symptoms represent the result of the infection process. Therefore, FER symptoms were assessed in sibling mutant and wild-type mature ears subjected to mock or *Fusarium* treatment. All inoculated genotypes showed higher FER incidence and severity as compared to mock-treated plants (**Supplementary Figures 4A, B**). Under our experimental conditions, the inoculum caused a severe infection in treated mature ears and no statistically significant differences were observed for both FER incidence and severity among genotypes. This is expected because *Fusarium* disease severity is determined also by other factors such as kernel-related resistance traits, besides those related to silk properties.

To test the susceptibility of each genotype to *Fusarium* spp. strains naturally present in the environment, FER incidence (**Supplementary Figure 4C**) and severity (**Supplementary Figure 4D**) were evaluated in open pollinated, non-treated plants. No differences in both fungal infection parameters were identified among genotypes.

Overall, our data indicate that silk cuticle composition may influence plant-fungus interaction resulting in changes in the fungal growth rate. Such changes were detected only during the early phases of *F. verticillioides* infection, while no differences were observed among genotypes at later phases, in which the fungus has infected the mature kernels. They also indicate that cuticle-related genes are responsive to *Fusarium* infection.

## 4 Discussion

### 4.1 The role of *ZmFDL1* and *ZmGL2* in controlling cuticular wax deposition on maize silks

Comparing mutant and wild-type plants, we established the role of *ZmFDL1* and *ZmGL2* in determining the composition of cuticular waxes on maize silks. Our work investigates the role of known cuticle-related genes in determining the abundance and composition of silk cuticular waxes. The *fdl1-1* mutant used in this study is the first isolated allele that allowed the characterization of this R2R3-MYB transcription factor (La Rocca et al., 2015), while the *gl2-ref* mutant refers to the *ZmGL2* gene (Bianchi et al., 1975; Tacke et al., 1995). Both *ZmGL2* and its paralog *ZmGL2-like* are members of the BAHD superfamily of acyltransferases with close sequence similarity to the *Arabidopsis thaliana AtCER2* gene (Xia et al., 1996; Alexander et al., 2020). *ZmFDL1* and *ZmGL2* have been located in the same co-expression network (Ma et al., 2017), although no evidence of *ZmGL2* differential expression was obtained in RNAseq experiments in which *fdl1-1* mutant and wild-type seedling transcriptomes were compared (Castorina et al., 2020; Liu X. et al., 2021). The roles of both genes were previously established in maize seedling leaves (Bianchi et al., 1975; Tacke et al., 1995; La Rocca et al., 2015; Alexander et al., 2020; Castorina et al., 2020), in which long-chain alcohols (69%), followed by aldehydes (25%), alkanes (4%) and esters (2%) are the main components of cuticular waxes (Javelle et al., 2010). Surface waxes on the stigmatic silks of maize are instead composed primarily of linear hydrocarbons, which comprise homologous series of alkanes, alkenes and dienes that are presumably biosynthesized by a series of parallel pathways of fatty-acid elongation and desaturation reactions, which are followed by sequential reduction and decarbonylation (Perera et al., 2010). Another relevant difference in silk cuticular wax composition consists in the trace accumulation to absence of alcohols or aldehydes (Loneman et al., 2017; Dennison et al., 2019; **Figures 1**-**4**). As was observed in previous studies (Perera et al., 2010; Loneman et al., 2017; Dennison et al., 2019), cuticular wax accumulation patterns in this study differ between the emerged and husk-encased portions of the silks, particularly that cuticular wax accumulation is higher in the emerged portions of silks and this is observed consistently for wildtype, as well as *fdl1*-*1* and *gl2-ref* plants. The increases in cuticular waxes observed for the emerged portions of silks may be attributed to changes in environment and exposure to adverse environmental conditions. Indeed, many key stress-response genes exhibit increased gene expression in emerged as compared to husk-encased portions of silks (McNinch et al., 2020).

In seedlings, the *fdl1-1* mutation causes a decrease in the primary cuticular wax components (Castorina et al., 2020). Similarly, lack of ZmFDL1 action in silks generates a reduction of the total wax load (**Figure 1**) due to the decreased accumulation of a wide number of compounds, including alkanes and alkenes of 20 carbons or greater (**Figure 2**). This general perturbation in cuticular wax compounds (**Figure 2**) may indicate that ZmFDL1 retains a regulatory role in silks, which might be exerted across the entire wax biosynthesis pathway, particularly in hydrocarbon synthesis.

Two realistic hypotheses can be formulated to interpret the link between the mutant wax phenotype on *fdl1-1* mutant silks and the variations in gene expression levels of *ZmCER1*, *ZmCER4* and *ZmWSD11* (**Supplementary Figure 3**). The first hypothesis considers that the cuticular wax alterations in *fdl1-1* could be due to defects in the elongation of the very long-chain acyl-CoA wax precursors. However, statistical differences were observed only for the unsaturated C18 LCFA on the emerged portions of the silks from *fdl1-1* as compared to WT, but not for other LCFAs (i.e. other saturated and unsaturated fatty acids of 16 carbon and 18 carbon chain lengths) and VLCFAs (i.e. saturated fatty acids of 20 and 22 carbon chain lengths). In the second hypothesis, the observed decrease of cuticular alkanes and alkenes could depend on genes directly involved in the synthesis of these specific metabolites. In both scenarios, decreases in long-chain alkanes and alkenes could promote a compensatory change in expression of cuticular wax biosynthesis genes in the *fdl1-1* mutant silks, as compared to the wild-type control. The increase in transcript level of *ZmCER1*, homolog of *AtCER1* (Bourdenx et al., 2011), putatively involved in the hydrocarbon forming complex and responsible for the conversion of aldehydes into alkanes (Bernard et al., 2012; Pascal et al., 2019), could be interpreted as an attempt to balance the decrease in the abundance of alkanes and alkenes. Similarly, the reduction in the expression level of the *ZmCER4* gene, homolog of *AtCER4* (Rowland et al., 2006), putatively encoding a fatty acid reductase that produces very long-chain primary alcohols from very long-chain acetyl-CoAs, could permit these substrates to remain available for the hydrocarbon-forming pathway. These results support the hypothesis that ZmFDL1 retains a regulatory role in silks. However, differently from what has been observed in seedlings, the expression of the *ZmWSD11* gene was not affected. *ZmWSD11*, the homolog of AtWSD11 (Takeda et al., 2013), is located downstream to *ZmCER4* in the alcohol forming pathway and putatively involved in the formation of long-chain wax esters.

Further analyses are needed to clarify the mechanism of action of *ZmFDL1* in silks. ZmFDL1 may take part in a broader, silk-specific regulatory complex, which exerts a distinct action in controlling the structural genes involved in the wax biosynthetic pathways, different from that one carried out in seedling leaves.

Changes caused by the lack of *gl2-ref* function seem to be more evident in seedlings than in silks (**Figures 3**, **4**). Only small changes in cuticular wax composition were observed on maize silks from *gl2-ref* mutant plants, particularly changes in specific alkenes, dependent on the position of the double bond, (**Figure 4**) and these changes do not impact the cuticular wax ultra-structure (**Figure 5**). In contrast, the cuticular waxes of *gl2-ref* seedlings exhibit a more obvious change in composition, particularly a reduction of C32 long-chain fatty acids, C32 primary alcohol, C32 aldehydes, C30 and C31 alkanes and a concomitant increased accumulation of shorter-chain compounds of the same categories: C28 long-chain fatty acids, C28 aldehydes, C27 alkanes (Alexander et al., 2020). This data, together with the observation that *ZmGL2* can complement a mutation in the corresponding *AtCER2* gene in Arabidopsis, demonstrates a role of *ZmGL2* in the elongation of fatty acids, by an as of yet undefined mechanism. The *ZmGl2* product might interact with the Fatty Acid Elongation (FAE) complex, as shown for AtCER2, and promote the accumulation of hydrocarbons of chain lengths longer than 28 carbons (Alexander et al., 2020). It is conceivable that this role is carried out in silks as well, and we propose that in this organ ZmGL2 differentially affects the synthesis of longer saturated and unsaturated alkyl-lipids. Further, it is possible that the lost function of *ZmGL2* in *gl2-ref* mutant plants is masked by a functional *ZmGL2-like* homolog that shares 63% amino acid similarity and is expressed in maize silks (Walley et al., 2016).

As evidenced by scanning electron microscopy, epicuticular waxes are distributed as an amorphous film on the silk surface (**Figure 5**) and crystalloids, which were detected on seedling leaves (Beattie and Marcell, 2002), are not formed. Despite the presence of changes in silk chemical composition of both *fdl1-1* and *gl2-ref* mutant genotypes compare to wild-type plants, it seems that, at the microscopic level, there is no effect on the morphology of the silk epicuticular layer.

### 4.2 Effect of changes in wax composition on silk-*Fusarium* interaction

Mutants in the *ZmFDL1-*encoding transcription factor and the fatty acid elongation pathway *ZmGL2* gene provide helpful genetic resources to study the involvement of silk cuticular waxes in modulating *F. verticillioides* growth. Studies conducted on *AtMYB96*, the *Arabidopsis thaliana* homolog of *ZmFDL1*, showed that mutants in this regulatory gene displayed higher cuticular permeability and a spontaneous accumulation of reactive oxygen species (ROS), even in the absence of the pathogen, resulting in increased resistance to *Botrytis cinerea* (Benikhlef et al., 2013). Also, other Arabidopsis mutants with increased cuticle permeability display strong resistance to pathogenic fungi (Bessire et al., 2007; Inada and Savory, 2011), which in turn is due to the constitutive production of ROS (L'Haridon et al., 2011). In maize, some genes involved in plant immune system pathways were shown to be up-regulated in *fdl1-1* mutant seedlings (Castorina et al., 2020). The *CHITINASE A1* (*ZmCTA1*; Zm00001d003190), the *CHITINASE B1* (*ZmCTB1*; Zm00001d025753), the *CHITINASE2* (*ZmCHN2*; Zm00001d036370) and the *CHITINASE27* (*ZmCHN27*; Zm00001d048903) genes have been shown to be involved in the resistance of maize to ear rot pathogen *Aspergillus flavus* (Hawkins et al., 2015) and were up-regulated by 5.3-, 4.2-, 3.0-, and 1.9-fold in *fdl1-1* seedlings. Moreover, the *RESPIRATORY BURST OXIDASE4* (*ZmRBOH4*; Zm00001d052653) gene, encoding for NADPH-oxidase, involved in ROS production and resistance to pathogens (Zhang et al., 2023), was up-regulated by 4-fold. In addition, specific pathogenesis-related genes (i.e. PR genes), including *ZmPR5* and *ZmPR3*, were up-regulated in *fdl1-1* seedlings. For example, the *PATHOGENESIS RELATED PROTEIN5* (*ZmPRP5*; Zm00001d031158) gene encodes for a defense-related protein with potential fungi toxic activity (Hu and Reddy, 1997; Zhang et al., 2018) and was up-regulated by 4-fold. The *PATHOGENESIS-RELATED PROTEIN3* (*ZmPRP3*; Zm00001d048947) gene is associated with early-stage resistance to *Aspergillus flavus* in maize (Liu H. et al., 2021) and was up-regulated by 1.5-fold. Interestingly, the activation of genes encoding PR proteins was observed in maize following infection by *F. verticillioides* (Lanubile et al., 2017). A link between cuticle changes ascribable to a mutant in the *AtCER2* gene, homologue of the maize *ZmGL2* gene, and variations in the expression of *PATHOGENESIS-RELATED 1* (*AtPR1*), a marker of the systemic acquired resistance (SAR) pathway, had been previously shown in *Arabidopsis thaliana* (Garbay et al., 2007). The *AtPR1* expression level decreased by two orders of magnitude in the *cer2* mutant, when compared to the wild-type plants (Garbay et al., 2007). In maize, the expression of *ZmPR1*, was shown to be induced during the interaction with *F. verticillioides* (Lanubile et al., 2012; Lanubile et al., 2014).

The involvement of the cuticle in the response to *F. verticillioides* infection is supported by the observation obtained in our work that the treatment with *Fusarium* induces changes in the transcript levels of *ZmFDL1* and *ZmGL2* genes (**Figure 7**). We then investigated the effect of alterations in silk cuticular waxes on the interaction with the ear rot pathogenic fungus, *F. verticillioides*. We considered that differences in waxes could affect silk-fungus interaction and therefore hyphal growth. In previous studies, genotypes resistant to *F. graminearum* displayed a thicker cuticle than susceptible genotypes and wax load was at least a component, if not the main component, of silk resistance to this fungus (Bergvinson and Reid, 1995). The conidia germination time and hyphal growth rate on silks is an important component of plant-pathogen interaction and may impact maize resistance to *F. graminearum.* In resistant maize genotypes, the fungus exhibited a longer time interval to access the kernels (Miller et al., 2003).

*F. verticillioides* growth was shown to be significantly higher on the silks of the *gl2-ref* mutant compared to its wild-type control siblings. The increased growth rate of *F. verticillioides* (**Figure 6**), could be correlated to the presence or absence of some lipid-constituents in the cuticular waxes, which eventually could lead to a change in the rate of fungus growth. It was previously shown that monomers derived from the hydrolysis of the cuticle induce a response in pathogens, and it was demonstrated that they can activate fungal development *in vitro* (Ahmed et al., 2003). In addition, surface waxes activate developmental processes in fungi. It is hypothesized that pathogens carry out a preliminary degradation of the cuticle to determine whether the conditions are favourable for penetration into the host tissues (Serrano et al., 2014). The higher susceptibility of wax-deficient mutants to pathogen attack may be the consequence of changes in cuticular waxes combined with a lack of basal expression levels of some defense-related genes, which normally keep the defense system primed in the absence of pathogen attacks. Because only small changes in cuticular wax composition were observed for *gl2-ref* silks, these very specific changes in the accumulation of specific alkanes and alkenes may be important for fungal-silk cuticle interactions. Further exploration of *gl2; gl2-like* double mutants in the future would provide insights into the role of the cuticle in resistance to *F. verticillioides* infection.

Differently from what was observed for *gl2-ref* silk, *F. verticillioides* had a similar growth rate on the silks of both wild-type and *fdl1-1* mutant plants, even with appreciable differences in silk cuticle composition in the *fdl1-1* mutant (**Figure 6**). We may thus speculate that a defective cuticle in *fdl1-1* mutant could allow a faster perception of pathogen-derived molecular determinants (i.e. pathogen-associated molecular patterns, PAMPs), thus resulting in unchanged resistance despite the wax defects. Another hypothesis is based on previous observations that cuticle monomers constitute a signal for the activation of the defense mechanisms, as an endogenous non-specific elicitor or DAMP (damage-associated molecular pattern) (Serrano et al., 2014). In *fdl1-1* mutant silks cuticle precursors could be over-accumulated due to incomplete synthesis of cuticle polymers, thus causing constitutive activation of the immune plant system. Overall, our data corroborate the hypothesis of an active role of the cuticle, which behaves not only as a physical barrier, but likely plays a more complex role during plant-pathogen interactions and in the plant innate immunity.

## Data availability statement

The original contributions presented in the study are included in the article/**Supplementary Material**. Further inquiries can be directed to the corresponding author.

## Author contributions

GCo and MDY-N designed the study and wrote the manuscript. GCa performed molecular quantification of Fusarium growth and gene expression analysis, graphical elaboration of the data and statistical analysis and wrote sections of the manuscript. MB and TH also performed statistical analyses and graphing. MI and GV supervised experiments and data analysis related to fungus treatment. GV, SS and MZ carried out field experiments and analyzed FER parameters with GCa. EC performed SEM analysis. All authors contributed to manuscript revision and approved the submitted version.
