# Supplementary material for: Roles of the MYB94/FUSED LEAVES1 (ZmFDL1) and GLOSSY2 (ZmGL2) genes in cuticle biosynthesis and potential impacts on Fusarium verticillioides growth on maize silks
Source: Front Plant Sci. 2023 Jul 20;14:1228394. doi: 10.3389/fpls.2023.1228394 (PMC10399752; doi:10.3389/fpls.2023.1228394)
Supplement: Supplementary file 1 [file DataSheet_1.pdf]

Supplementary Material

Roles of the *MYB94/FUSED LEAVES1 (ZmFDL1)* and *GLOSSY2 (ZmGL2)* genes in cuticle biosynthesis and potential impacts on *Fusarium verticillioides* growth on maize silks

Giulia Castorina[1], Madison Bigelow[2], Travis Hattery[2], Massimo Zilio[1], Stefano Sangiorgio[1], Elisabetta Caporali[3], Giovanni Venturini[1], Marcello Iriti[1, 4], Marna D. Yandeau-Nelson[2, *], Gabriella Consonni[1, *].

*** Correspondence:**

Corresponding Author: gabriella.consonni@unimi.it

Co-corresponding Author: myn@iastate.edu

1 Supplementary Data

The Supplementary Materials including Supplementary Figures 1–4, Supplementary Tables 1–4.

2 Supplementary Figures and Tables

Supplementary Figure File | The figures include: i) Experimental inoculation of maize silks with *Fusarium verticillioides*; ii) standard curve to set the molecular quantification of fungal growth on maize silks; iii) cuticle-related gene expression in *fdl1-1* silks; iv) FER incidence and FER severity in wild-type, *fdl1-1* and *gl2-ref* ears.

Supplementary Data Sheet 1| The data includes 4 sheets, i) the list of primers used in this study; ii) the list of cuticular wax metabolites in wild-type and *fdl1-1* mutant silks; iii) the list of cuticular wax metabolites in WT and *gl2-ref* mutant silks; iv) Scheme of cuticular wax composition of WT, *fdl1-1* and *gl2-ref* plants in different organs.

2.1 Supplementary Figures

Supplementary Figure 1. Experimental inoculation of maize silks with *Fusarium verticillioides*. Inoculations were performed by spraying the maize silks with a suspension of *F. verticillioides* conidia (A). To promote conidia germination, each maize ear was covered with a transparent plastic bag to

maintain high humidity (B). To quantify fungal growth, whole silks were collected at 0 and 72 hours after inoculation (C). The plastic bags were removed from the ears that were intended for visual quantification of ear rot disease symptoms, and (D) replaced with a closed-mesh wire bag.

Supplementary Figure 2. Molecular quantification of *Fusarium verticillioides*. Serial dilutions of fungal DNA, extracted from lyophilized pure culture, were (A) first amplified by PCR for the *FvFUM1* gene and then (B) by qPCR to generate a standard curve to eventually quantify fungal growth on maize silks. In panel B, values represent the mean fold change of three independent technical replicates. Error bar ±SE.

Supplementary Figure 3. FDL1 regulates the expression of cuticle-related genes in silk. Gene expression level of putative cuticle-related genes, analyzed by RT-qPCR, in *fdl1-1* and wild-type (WT) control plants. Values represent the mean fold change variations of four biological replicates. Error bar ±SD. Comparison is made between wild-type and homozygous *fdl1-1* genotypes. Significant difference was assessed by Student's t-test (**P, 0.01; ns, not significant).

Supplementary Figure 4. Analysis of *Fusarium verticillioides* infection. Fungal disease symptoms were measured through the FER incidence (A) and the FER severity (B) parameters in wild-type (WT), *fdl1-1* and *gl2-ref* genotypes after field experimental inoculations to evaluate the incidence and magnitude of the infection, respectively. The environmental *Fusarium verticillioides* contamination (C) of open pollinate ears has been also evaluated as FER incidence (C) and FER severity (D). Values represent the average ± SE of 12 (A, B) and 30 (C, D) independent biological replicates. Error bar ±SE. Significant differences between genotypes were assessed by two-way ANOVA.

2.2 Supplementary Tables

Supplementary Table 1. List of primers.

Supplementary Table 2. Cuticular wax composition of silks from WT and *fdl1-1* homozygous mutant plants. ND, Not Detected

Supplementary Table 3. Cuticular wax composition of silks from WT and *gl2-ref* homozygous mutant plants. ND, Not Detected

Supplementary Table 4. Cuticular waxes on different organs. Comparison of total wax load (wax), long chain fatty acids (FAs), very-long-chain fatty acids (VLCFAs), aldehydes, alkanes and alkenes on both seedlings and silks of *fdl1-1* and *gl-ref2* mutants with their relative wild-type (WT) control plants. Significative differences are reported with the minus sign: (-) indicates a reduction equal to or lower than 50% and (- -) indicates a reduction higher than 50%. nd = not detected. Differences in seedlings are reported from previously published data (Castorina et al., 2020; Alexander at el., 2020).
