# Supplementary material for: Roles of the MYB94/FUSED LEAVES1 (ZmFDL1) and GLOSSY2 (ZmGL2) genes in cuticle biosynthesis and potential impacts on Fusarium verticillioides growth on maize silks
Source: Front Plant Sci. 2023 Jul 20;14:1228394. doi: 10.3389/fpls.2023.1228394 (PMC10399752; doi:10.3389/fpls.2023.1228394)
Supplement: Supplementary file 2 [file DataSheet_2.pdf]

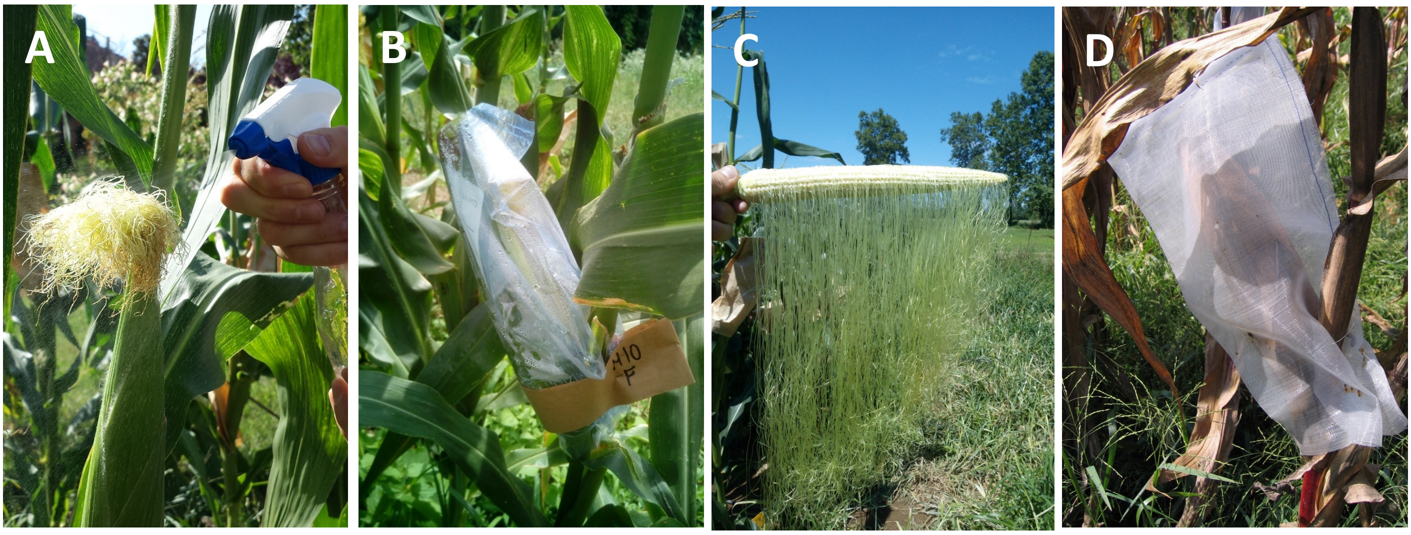

Supplementary Figure 1. Experimental inoculation of maize silks with *Fusarium verticillioides*. Inoculations were performed by spraying the maize silks with a suspension of *F. verticillioides* conidia (A). To promote conidia germination, each maize ear was covered with a transparent plastic bag to maintain high humidity (B). To quantify fungal growth, whole silks were collected at 0 and 72 hours after inoculation (C). The plastic bags were removed from the ears that were intended for visual quantification of ear rot disease symptoms, and (D) replaced with a closed-mesh wire bag.

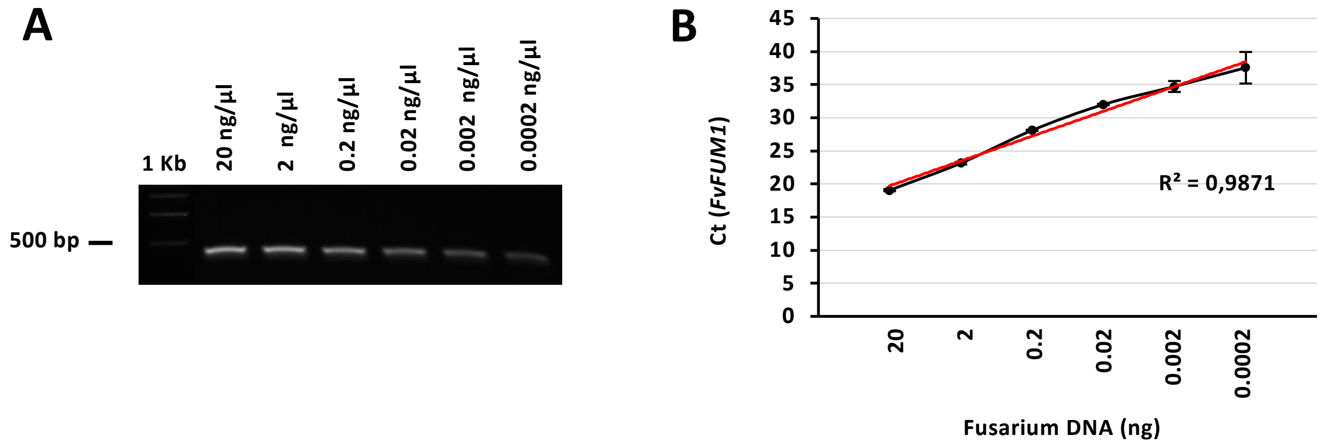

Supplementary Figure 2. Molecular quantification of *Fusarium verticillioides*. Serial dilutions of fungal DNA, extracted from lyophilized pure culture, were (A) first amplified by PCR for the *FvFUM1* gene and then (B) by qPCR to generate a standard curve to eventually quantify fungal growth on maize silks. In panel B, values represent the mean fold change of three independent technical replicates. Error bar ±SE.

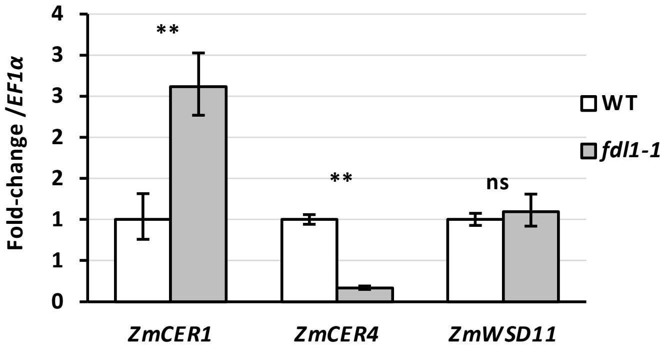

Supplementary Figure 3. FDL1 regulates the expression of cuticle-related genes in silk. Gene expression level of putative cuticle-related genes, analyzed by RT-qPCR, in *fdl1-1* and wild-type (WT) control plants. Values represent the mean fold change variations of four biological replicates. Error bar ±SD. Comparison is made between wild-type and homozygous *fdl1-1* genotypes. Significant difference was assessed by Student's t test (**P , 0.01; ns, not significant).

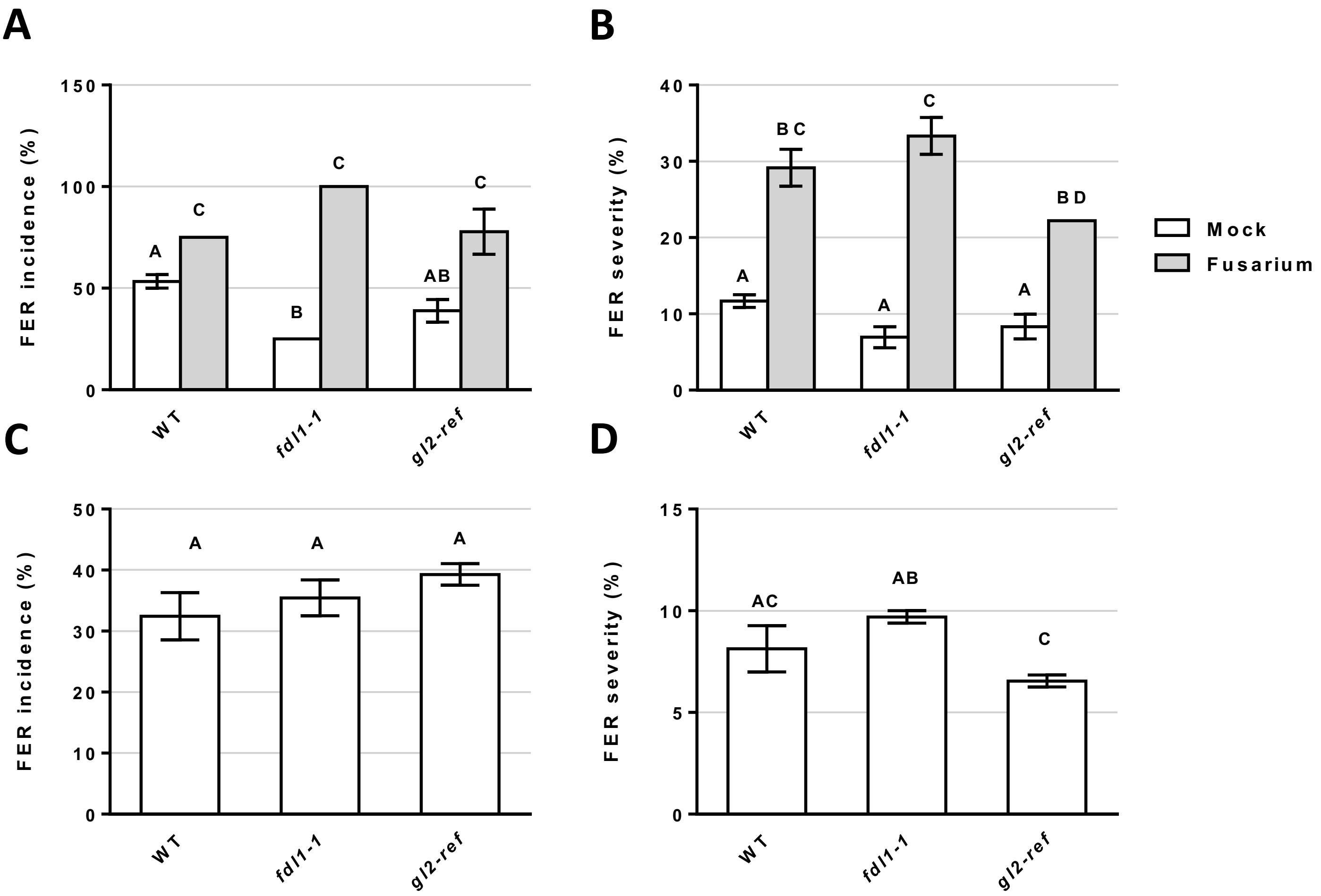

Supplementary Figure 4. Analysis of *Fusarium verticillioides* infection. Fungal disease symptoms were measured through the FER incidence (A) and the FER severity (B) parameters in wild-type (WT), *fdl1-1* and *gl2-ref* genotypes after field experimental inoculations to evaluate the incidence and magnitude of the infection, respectively. The environmental *Fusarium verticillioides* contamination (C) of open pollinate ears has been also evaluated as FER incidence (C) and FER severity (D). Values represent the average ± SE of 12 (A, B) and 30 (C, D) independent biological replicates. Error bar ±SE. Significant differences between genotypes were assessed by two-way ANOVA.
